# Long-term effects of anti-reflux surgery on the physiology of the esophagogastric junction

**DOI:** 10.1007/s00464-015-4144-7

**Published:** 2015-03-19

**Authors:** Boudewijn F. Kessing, Albert J. Bredenoord, Marlies P. Schijven, Donald L. van der Peet, Mark I. van Berge Henegouwen, André J. P. M. Smout

**Affiliations:** 1 Department of Gastroenterology and Hepatology, Academic Medical Center, Meibergdreef 9, 1105 AZ Amsterdam, The Netherlands; 2 Department of Surgery, Academic Medical Center, Amsterdam, Netherlands; 3 Department of Surgery, VU University Medical Center, Amsterdam, Netherlands

**Keywords:** GERD, Fundoplication, pH monitoring, Esophagus

## Abstract

**Background:**

Studies performed shortly after anti-reflux surgery have demonstrated that the reduction of reflux episodes is caused by a decrease in the rate of transient lower esophageal sphincter relaxations (TLESRs) and a decrease in the distensibility of the esophagogastric junction (EGJ). We aimed to assess the long-term effects of surgical fundoplication on the physiology of the EGJ.

**Methods:**

We included 18 patients who underwent surgical fundoplication >5 years before and 10 GERD patients who did not have surgery. Patients underwent 90-min combined high-resolution manometry and pH-impedance monitoring, and EGJ distensibility was assessed.

**Results:**

Post-fundoplication patients exhibited a lower frequency of reflux events than GERD patients (2.0 ± 0.5 vs 15.1 ± 4.3, *p* < 0.05). The rate of TLESRs (6.1 ± 0.9 vs 12.6 ± 1.0, *p* < 0.05) and their association with reflux (28.3 ± 9.0 vs 74.9 ± 6.9 %, *p* < 0.05) was lower in post-fundoplication patients than in GERD patients. EGJ distensibility was significantly lower in post-fundoplication patients than in GERD patients. Recurrence of GERD symptoms after fundoplication was not associated with an increased number of reflux episodes, nor was it associated with an increased distensibility of the EGJ or an increase in the number of TLESRs.

**Conclusion:**

More than 5 years after anti-reflux surgery, patients still exhibit a lower rate of TLESRs and a reduced distensibility of the EGJ compared with medically treated GERD patients. These data suggest that the effects of surgical fundoplication on EGJ physiology persist at the long term and underlie the persistent reduction of reflux events.

The esophagogastric junction (EGJ), formed by the lower esophageal sphincter (LES) and the crural diaphragm, prevents gastric content from entering the esophagus [1]. In patients suffering from gastroesophageal reflux disease (GERD), this barrier is compromised and reflux of gastric content can occur more freely causing symptoms (heartburn, regurgitation) as well as damage to the esophagus (esophagitis) [2]. Most reflux episodes occur during a so-called transient relaxation of the lower esophageal sphincter (TLESR) [3] (Fig. 1). These are spontaneous sphincter relaxations that are not induced by swallowing [4].

**Fig. 1:**
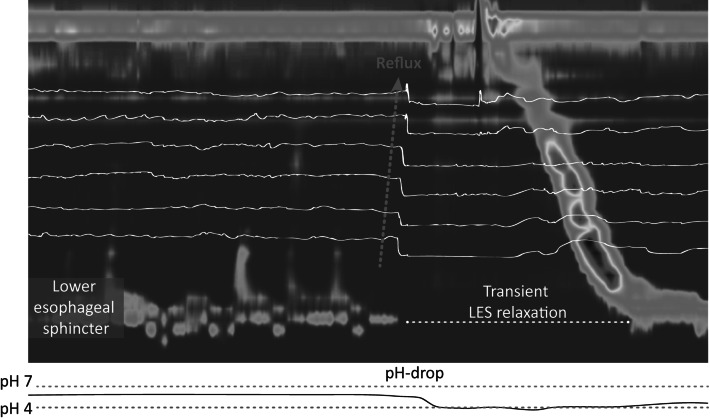
Example of a transient lower esophageal sphincter relaxation (TLESR) which is associated with a reflux episode as measured by combined high-resolution manometry and pH-impedance monitoring. The continuous high-pressure zone of the lower esophageal sphincter is temporarily interrupted during which a reflux episode can occur. The *dotted arrow* indicates the direction of reflux. The *white lines* represent impedance channels and the reflux episode is characterized by a drop in impedance starting in the distal channel. The pH-channel is represented by the most lower graph and demonstrates an acidic reflux episode

Initial management in GERD patients is medical therapy using proton pump inhibitors (PPI) [5]. In patients who are intolerant for PPI therapy or patients in whom troublesome symptoms persist despite PPI therapy, anti-reflux surgery is indicated [5]. The short-term effects of the surgical fundoplication are good, resulting in excellent symptomatic and objective outcomes such as a reduction in the number of reflux episodes. Broeders et al. [6] reported that 10 years after anti-reflux surgery, as many as 25–28 % of the patients has become daily PPI-dependent again and/or has recurrent reflux symptoms. Surprisingly, abnormal esophageal acid exposure was found to be present in only 20–30 % of these subjects, which suggests that a concomitant functional disease such functional dyspepsia or functional heartburn is the most frequent cause of recurrent symptoms during the long-term follow-up after a fundoplication [6].

The exact mechanisms through which anti-reflux surgery prevents gastroesophageal reflux are not completely clear. However, it has been proposed that three main mechanisms play a role. The first of these is anatomical restoration of the EGJ by the repair of a hiatal hernia, if present. Secondly, anti-reflux surgery reduces the rate of TLESRs as well as the proportion of TLESRs that are associated with acid reflux [7]. The third proposed mechanism is decreased distensibility of the EGJ [8, 9]. It should be noted that the studies that demonstrated the latter two physiological effects were carried out shortly after fundoplication. The long-term effects of fundoplication on the physiology of the EGJ are still unknown.

We hypothesized that in patients who underwent a surgical fundoplication >5 years ago, the rate of TLESRs and the distensibility of the EGJ are still decreased compared with GERD patients without a fundoplication.

We therefore aimed to assess the long-term effects of surgical fundoplication on the physiology of the EGJ by studying the rate of TLESRs and distensibility of the EGJ.

## Methods

### Subjects

All patients who underwent a laparoscopic fundoplication in the Academic Medical Center Amsterdam and the VU University Medical Center in Amsterdam between 1999 and 2008 were invited to participate in this study. Patients were eligible when they underwent their fundoplication >5 years before inclusion in this study. We excluded patients in whom a multiple operations were performed or when severe complications had occurred during the operation.

As a control group, we prospectively included ten patients matched for age and sex with GERD which we defined as an association between symptoms and reflux episodes during ambulatory 24-h pH-impedance monitoring. In these patients, we performed a measurement protocol similar to patients who underwent a fundoplication.

### Questionnaires

All subjects filled out the Reflux Disease Questionnaire (RDQ) to assess the severity of symptoms of GERD [10]. In summary, the RDQ provides a score for regurgitation and heartburn based on the severity and frequency of the symptoms. The RDQ scores range between 0 and 5, whereas subjects without complaints have a score of 0. Furthermore, the total RDQ score provides a score based on the severity and frequency of heartburn and regurgitation.

Furthermore, the recurrence of symptoms of GERD was assessed in all patients. If patients indicated that their initial symptoms of GERD had recurred, they were considered as patients with recurrent symptoms.

### EGJ distensibility

Subjects arrived to the clinic after an overnight fast (no fluid or food after midnight). EGJ distensibility was assessed using the EndoFLIP device. The EndoFLIP system for endoluminal impedance planimetry (Crosspon Inc, Carlsbad CA, USA) consists of a catheter (outer diameter 3 mm) with an infinitely compliant cylindrical bag at the distal tip and dedicated software and hardware. The balloon covers the distal 10 cm and contacts the esophageal wall upon filling with saline solution. A series of electrodes within the balloon measures the impedance at 16 longitudinal levels, and these measurements are used to calculate cross-sectional areas at these levels.

The catheter was introduced transnasally and positioned into the stomach. After inflation of the distal balloon, the catheter was pulled back and the balloon was placed across the EGJ. Each measurement consisted of volume-controlled distension with of 20, 30, 40, and 50 mL. The lowest cross-sectional area at different fluid volumes was considered as the diameter of the EGJ. Distensibility was calculated as the lowest cross-sectional area divided by pressure (mm^2^/mmHg).

Distensions were stopped if the patient indicated discomfort.

### HRM and pH-impedance monitoring

Catheters for high-resolution manometry (HRM) and impedance measurement (see below) were inserted transnasally after the EndoFLIP catheter had been removed. Combined HRM and impedance monitoring was performed for 90 min immediately after ingestion of a standardized meal consisting of two pancakes (140 kcal, 23.4 g carbohydrate, 1.6 g fat).

The impedance monitoring catheter was fitted with six impedance recording segments and one ISFET pH electrode (Unisensor AG, Attikon, Switzerland). Impedance recording segments were located at 2–4, 4–6, 6–8, 8–10, 14–16, and 16–18 cm above the upper border of the LES. A sample frequency of 50 Hz was used to record impedance signals. Concurrently, HRM was carried out using a solid-state HRM assembly with 36 solid-state sensors spaced at 1-cm intervals (Unisensor AG, Attikon, Switzerland). The manometry catheter was positioned to record from hypopharynx to stomach. A sampling frequency of 20 Hz was used to record HRM signals. Impedance and pressure signals were recorded and automatically synchronized on a personal computer system [Medical Measurement Systems (MMS), Enschede, the Netherlands]. Before the onset of the measurement, the HRM pressure tracings were zeroed to the atmospheric pressure.

The occurrence of TLESRs was analyzed according to the modified criteria published by Holloway et al. [11]. Impedance tracings were analyzed for the occurrence of reflux episodes according to previously published criteria [12]. The presence of a double high-pressure zone as observed during high-resolution manometry was assessed [13]. A double high-pressure zone is an indication of two separate pressure zones caused by the diaphragm and the LES and occurs in patients with hiatal hernia. The occurrence of a double high-pressure zone after fundoplication therefore suggests that a hiatal hernia is present and could, in theory, contribute to the occurrence of reflux episodes.

### Statistical analysis

Throughout the manuscript, data are presented as mean ± SEM. Statistical analysis was performed using Prism software version 5 (GraphPad, La Jolla CA, USA). Comparisons were analyzed using the Students *t* test. Differences were considered statistically significant when *p* < 0.05.

## Results

### Subjects

A total of 18 patients [mean age 59 (range 43–73), 11 male] in whom a fundoplication was performed >5 years ago successfully completed the measurement protocol. A total of 13 out of 18 patients underwent a laparoscopic Nissen fundoplication and five out of 18 patients underwent a Toupet fundoplication. A total of eight out of 18 patients in whom a fundoplication was performed reported that their preoperative reflux had returned. Therefore, these eight patients were considered to be patients with recurrent symptoms.

In patients who underwent a fundoplication, the RDQ score was significantly lower compared with GERD patients without a fundoplication, as were the scores for regurgitation and heartburn (Table 1).

**Table 1:** Outcomes of symptom questionnaires in patients who underwent the surgical fundoplication >5 year ago and GERD patients without a fundoplication

	Post-fundoplication (*n* = 18)	GERD without fundoplication (*n* = 10)	*p* value
Heartburn	1.5 ± 1.6	3.2 ± 1.4	<0.05
Regurgitation	0.5 ± 1.0	3.1 ± 1.6	<0.001
Total RDQ score	1.1 ± 1.3	3.2 ± 1.3	<0.001

Results are presented as mean ± SEM

### EGJ distensibility measurements

EndoFLIP measurements were completed by all GERD patients without a fundoplication and were stopped during the measurement due to severe discomfort in one post-fundoplication patient. EGJ distensibility was significantly higher in GERD patients who did not undergo a fundoplication compared with patients post-fundoplication at 20, 30, and 40 mL (Table 2). Balloon pressure was significantly higher in patients who underwent fundoplication compared with GERD patients without fundoplication at 20 mL (14.5 ± 1.6 vs 9.3 ± 1.5, *p* < 0.05), 30 mL (19.9 ± 2.0 vs 13.4 ± 1.6, *p* < 0.05), 40 mL (30.2 ± 2.5 vs 21.3 ± 2.5, *p* < 0.05), but not at 50 mL (40.3 ± 3.2 vs 32.4 ± 2.9, NS). Differences in cross-sectional area of the EGJ did not reach statistical significance at the different balloon volumes.

**Table 2:** Results of the combined HRM and pH-impedance measurements and EndoFLIP measurements in patients who underwent surgical fundoplication >5 year ago and GERD patients without a fundoplication

	Post-fundoplication (*n* = 18)	GERD without fundoplication (*n* = 10)	*p* value
Number of reflux episodes/90 min	2.0 ± 0.5	15.1 ± 4.3	<0.05
Number of TLESRs/90 min	6.1 ± 0.9	12.6 ± 1.0	<0.05
Percentage of TLESRs with reflux	28.3 ± 9.0	74.9 ± 6.9	<0.05
Distensibility (mm^2^/mmHg)			
50 mL	6.2 ± 1.2	8.0 ± 1.7	NS
40 mL	4.1 ± 0.6	7.1 ± 1.4	<0.05
30 mL	2.7 ± 0.4	5.0 ± 0.9	<0.05
20 mL	2.3 ± 0.4	4.0 ± 0.8	<0.05
End-exp LES pressure (mmHg)	11.5 ± 1.7	8.2 ± 3.0	NS

Results are presented as mean ± SEM

*TLESR* transient lower esophageal sphincter relaxation, *LES* lower esophageal sphincter, *NS* nonsignificant

### Combined pH-impedance monitoring and high-resolution manometry

Combined pH-impedance monitoring and HRM was completed by all subjects. However, in one post-fundoplication patient, the HRM signals showed an artifact which prohibited analysis of the measurement. In another patient of the fundoplication group, a technical problem prohibited analysis of the pH-impedance signals. In the latter, two patients only the HRM or pH-impedance signals were analyzed.

A double high-pressure zone was observed in seven out of 18 patients who underwent a fundoplication and was observed in six out of 10 GERD patients without a fundoplication. Resting pressure of the lower esophageal sphincter (respiratory minimum) was not significantly different between patients who underwent a fundoplication compared with GERD patients who did not undergo a fundoplication (Table 2). The number of TLESRs was significantly lower in patients who underwent a fundoplication compared with GERD patients without a fundoplication (6.1 ± 0.9 vs 12.6 ± 1.0, *p* < 0.05). Furthermore, the percentage of TLESRs that was associated with a reflux episode was significantly higher in patients with GERD compared with the post-fundoplication patients. Notably, in patients who underwent a fundoplication, reflux episodes occurred most frequently during a TLESR (71 %). Other mechanisms observed in these patients were swallow-induced reflux (16 %), free reflux (6 %), and reflux during abdominal pressure peaks such as strain-induced reflux and cough-induced reflux (6 %).

### Recurrence of GERD symptoms after fundoplication

A total of eight out of 18 patients were considered as having recurrent symptoms after fundoplication. Patients with recurrent symptoms had significantly higher RDQ scores for heartburn (2.9 ± 0.4 vs 0.4 ± 0.3, *p* < 0.05), regurgitation (1.3 ± 0.5 vs 0.0 ± 0.0, *p* < 0.001), and total RDQ score (2.2 ± 0.4 vs 0.2 ± 0.2, *p* < 0.001) compared with patients without recurrent symptoms. A double high-pressure zone was observed in two out of eight patients with recurrent symptoms and the latter was observed in five out of 10 patients without recurrent symptoms.

The number of reflux episodes reported during the 90-min measurement period was not significantly different between patients with and without recurrent symptoms after fundoplication (Table 3). Furthermore, we did not observe a difference in the number of TLESRs, nor did we observe a difference in the distensibility of the EGJ at different balloon volumes.

**Table 3:** Results of the combined HRM and pH-impedance measurements and EndoFLIP measurements in post-fundoplication patients with recurrent reflux symptoms compared with patients without recurrent symptoms

	Recurrent symptoms (*n* = 8)	No recurrent symptoms (*n* = 10)	*p* value
Number of reflux episodes/90 min	3.1 ± 0.9	1.0 ± 1.1	NS
Number of TLESRs/90 min	6.0 ± 1.5	6.1 ± 1.0	NS
Distensibility (mm^2^/mmHg)			
50 mL	4.7 ± 1.3	7.6 ± 1.8	NS
40 mL	3.3 ± 0.6	4.7 ± 0.9	NS
30 mL	2.5 ± 0.7	2.8 ± 0.6	NS
20 mL	2.7 ± 0.8	2.0 ± 0.3	NS

## Discussion

Fundoplication is a surgical intervention which has been applied extensively in the treatment of GERD over the last few decades. Excellent symptomatic and objective outcomes during short- and long-term follow-up have been reported [6, 14]. Since the mechanisms through which these beneficial effects are achieved had only been assessed in studies carried out shortly after fundoplication, our study aimed to assess the long-term effects of the fundoplication on the physiology of the EGJ.

Distensibility of the EGJ was found to be increased in patients with GERD [15]. It has been suggested that this increased distensibility contributes to an increased incidence of gastroesophageal reflux episodes [15]. Pandolfino et al. demonstrated that one of the short-term physiological effects of surgical fundoplication is reduced distensibility of the EGJ, and they have confirmed this finding using the EndoFLIP device [8, 9]. Therefore, decreased distensibility of the EGJ due to the creation of the surgical fundoplication is believed to be an important contributor to the reduction in reflux episodes. Our study clearly demonstrates that patients who underwent a surgical fundoplication >5 years ago still have a persistent markedly reduced distensibility of the EGJ. Therefore, this reduced distensibility of the EGJ after fundoplication appears to be an important physiological mechanism in the long-term reduction of reflux episodes.

In the beginning of the 1980s, TLESRs were recognized as the most important mechanism during which gastroesophageal reflux episodes occurred [16]. Straathof et al. [17] demonstrated that surgical fundoplication reduces the number of TLESRs. This effect of the fundoplication could be attributed to a reduced elicitation of TLESRs in response to gastric distension [18]. Furthermore, fundoplication not only reduces the number of TLESRs but also reduces the percentage of TLESRs which is associated with a reflux episode [7]. Therefore, the reduction in TLESRs and their association with reflux is considered to be an important physiological mechanism by which the fundoplication reduces the number of reflux episodes. However, the long-term effects of the fundoplication on the elicitation of TLESRs were hitherto unknown. In theory, during a long-term follow-up, the ability to trigger TLESRs could be restored, thereby re-allowing reflux episodes to occur during TLESRs. Our study clearly demonstrates that during a long-term follow-up after fundoplication, the rate of TLESRs is still markedly decreased compared with GERD patients without a fundoplication. Furthermore, the proportion of TLESRs associated with gastroesophageal reflux episodes was also clearly reduced. The results of our study therefore show that the reduced incidence of TLESRs and their reduced association with reflux episodes are important physiological mechanisms in the long-term reduction of reflux episodes after fundoplication.

Broeders et al. [6] demonstrated that during a long-term follow-up, patients with recurrent symptoms often do not exhibit pathological acid exposure times. Accordingly, we also did not observe a significant difference in the number of reflux episodes between patients with recurrent symptoms >5 years after fundoplication and patients without recurrent symptoms. In theory, 24-h pH-impedance monitoring could have identified such a difference, but these measurements were not performed in our study. However, our findings lend further support to the notion that “recurrent” symptoms after fundoplication are mostly caused by a concomitant functional disease such as functional heartburn or functional dyspepsia. Since we also observed similar numbers of reflux episodes in patients with recurrent symptoms and patients without recurrent symptoms, it is not surprising that we did not observe a difference in the number of TLESRs or in the distensibility of the EGJ. However, Broeders et al. also demonstrated that a small percentage of patients with recurrent symptoms do exhibit pathological acid exposure times. The limited number of patients included in our study made it impossible to identify this subgroup. Further studies are needed to explore the EGJ physiology in patients with true recurrence after fundoplication.

A limitation of this study is the inclusion of patient who underwent the Nissen fundoplication and the Toupet fundoplication. In theory, a difference could exist between these two surgical techniques. However, since the short-term physiological effects of the two techniques have many similarities, we believe that the long-term effects can be studied in these two groups together.

In conclusion, during a follow-up >5 years after fundoplication, the rate of TLESRs as well as their association with reflux episodes is reduced compared with patients with GERD who did not undergo a fundoplication. Furthermore, the distensibility of the EGJ is markedly decreased in patients >5 years after fundoplication compared with GERD patients who did not undergo a fundoplication. Our findings therefore suggest that the effects of the surgical fundoplication persist during a long-term follow-up and could underlie the long-term reduction in reflux episodes.
